# Correlation of sonographic and cytologic patterns of thyroid nodules

**DOI:** 10.11604/pamj.2021.39.220.28505

**Published:** 2021-07-29

**Authors:** Faosat olayiwola Jinadu, Zainab Odunaiya, Yemisi Oluseyi Kila Uvie-Emegbo, Abimbola Tawaqualit Ottun, Ayokunle Moses Olumodeji

**Affiliations:** 1 Department of Radiology, Lagos State University Teaching Hospital, Lagos, Nigeria,; 2 Department of Pathology, Lagos State University College of Medicine/ Lagos State University Teaching Hospital, Lagos, Nigeria,; 3 Department of Obstetrics and Gynaecology, Lagos State University College of Medicine/Lagos State University Teaching Hospital, Lagos, Nigeria,; 4 Department of Obstetrics and Gynaecology, Lagos State University Teaching Hospital, Lagos, Nigeria

**Keywords:** Ultrasound thyroid assessment, thyroid nodules, FNAC

## Abstract

**Introduction:**

thyroid nodules are palpable in about 8% of adults. It is necessary to differentiate benign nodules from malignant ones by the non-invasive ultrasonography thereby reducing the frequency of the invasive fine needle aspiration cytology (FNAC). The study assessed the sonographic and FNAC patterns of thyroid nodules for benign and malignant features in a black African population.

**Methods:**

this was a hospital-based, cross-sectional study design over a 1-year period in which one hundred and seven (107) consenting patients between 15 to 80 years of age with palpable thyroid masses by convenience sampling, were consecutively recruited to have both thyroid ultrasound scan and FNAC of their thyroid mass sequentially. Frequency, percentages and two-by-two contingency table were employed for data analysis.

**Results:**

the sonographic features of the thyroid nodules varied from round 80 (74.8%) to oval 25 (23.4%) masses, the presence of thin peripheral halo 83 (77.6%), heterogeneous echo-pattern 104 (97.2%) with cystic component and peripheral vascularity 75 (70.1%). One hundred and five (98.1%) study participants showed benign features on sonography while two had features suspicious of malignancy; however FNAC result in the same group of patients was suggestive of benign masses in all 107 (100%) patients. Histology however confirmed malignancy in the 2 participants with ultrasound features suggestive of malignant thyroid nodules.

**Conclusion:**

ultrasonography is very sensitive in the characterization of thyroid nodules into benign or suspicious for malignancy in black African population.

## Introduction

Thyroid disorders are the second most common endocrine disorders in Nigeria with a prevalence of 1.6% [1]. Nodular thyroid diseases are relatively common and found in 3-7% of the population worldwide [2]. The primary goal of thyroid nodule evaluation is to determine if a nodule is malignant or benign and whether it will or will not require surgery [1]. In contrast to the high prevalence of thyroid nodules, thyroid cancer is rare, as fewer than 7% of all nodules are malignant and it is critical that they be identified accurately [3]. The superficial location of the thyroid gland makes it suitable for high frequency sonography which is the imaging modality of choice [4]. The role of FNAC in evaluating euthyroid patient with thyroid nodule cannot be overemphasized as it reduces the unnecessary thyroidectomy for patients with benign nodules [5]. However, the combination of insufficient access to pathologists and the variable standards of pathology in sub-Saharan Africa undoubtedly mean that a significant proportion of cancer patients are receiving untimely diagnosis [6]. It was therefore expedient to compare thyroid ultrasonography with FNAC patterns in predicting benign or suspicious thyroid masses in an African population.

## Methods

**Study design and setting**: a cross-sectional research design was used to conduct the study in the Lagos State University Teaching Hospital, Lagos, Nigeria over a 1 year period (January 2016 to January 2017).

**Study population**: one hundred and seven consenting patients with palpable thyroid mass/masses, of both sexes and aged 15-80 years, presenting at the study hospital for treatment were recruited into the study using convenience sampling. Excluded from the study were patients less than 15 years of age, patients who had surgery/radiotherapy of the thyroid gland, pregnant women, patients who had thyroid FNAC done prior to the scan, patients on thyroid medications or radioactive iodine as well as non-consenting patients.

**Study procedure**: a General Electric LOGIC 5 expert ultrasound machine with colour flow Doppler with a 10MHz linear probe made in the USA, (2003) set to the 2D mode was used for the thyroid examination. During thyroid sonography, the patient was placed in the supine position, on the examination couch and the neck slightly extended with the aid of a sandbag placed under the patient´s shoulders and face turned away from the side being scanned. A coupling gel was applied to the anterior portion of the neck to obliterate the intervening airspace. A high frequency linear probe (10Hz) was used to scan both lobes of the thyroid gland in the axial and longitudinal planes. The size, shape, margin and echo-texture of the masses were documented. The masses were checked for calcifications and colour flow Doppler was employed to assess blood flow. Where the masses were multiple, the largest was measured in transverse and longitudinal planes and assessment of the different features of the masses were also made.

The transducer was then moved in the supero-inferior and medio-lateral directions until both lobes of the thyroid gland, the left and right common carotid arteries and the adjacent internal jugular vein were seen. Any mass or masses seen was assessed for size, shape, echo-texture, margin, lymphadenopathy, infiltration into adjacent muscles, and calcification. Vasculature of all nodules was also noted. The thyroid nodules were labelled benign or suspicious of malignancy. On Doppler interrogation the colour flow pattern was labelled a vascular, peripheral, peripheral with central vascularity, predominantly central with peripheral flow and central vascularity. USS features of benignity include cystic/predominantly cystic composition, wider than tall shape, regular well defined margins, heterogenous echopattern and thin peripheral halo [7]. USS features suspicious of malignancy include micro-calcifications, local invasion, lymph node metastases, nodule that is taller than it is wide and markedly reduced echogenicity [8-10]. Other features, such as the absence of a halo, ill-defined irregular margins, solid composition, extreme hypoechogenicity and vascularity, are less specific but may be useful ancillary signs [8-10]. The Doppler colour flow pattern was categorized from previous studies done. Flow patterns were classified as 0-4: Type 0 - No visible flow; Type 1 -Peripheral flow only; Type 2 - Peripheral flow with small amount of central flow; Type 3 -Peripheral with extensive central flow; Type 4 - Central flow only [11,12]. Nodules with types 0 - 2 flow pattern were categorized as benign. Nodules with types 3 and 4 flow patterns were categorized as suspicious for malignancy [11,12].

All participants then had FNAC of the thyroid masses by pathologists in the Department of Pathology, Lagos State University Teaching Hospital. They were requested to remove clothing and jewellery from the neck and sat in an upright position in a chair. The procedure (FNAC) was not ultrasound guided. A 23 gauge needle attached to a 10ml syringe was used for two to three aspirations on each nodule. For each nodule, samples were taken in a clockwise pattern roundabout and within the nodules. With multiple nodules the largest or most suspicious was aspirated based on the location on the ultrasound report. Cytology smear was made on six slides. Three slides were air dried for quick staining while the remaining three were fixed with 95% alcohol and stained with Papanicolau stain. The slides were reported according to the Bethesda system for reporting thyroid cytology [13]. Cytology results were then compared to the USS findings. Participants with suspicion of malignancy on ultrasound and/or FNAC had thyroidectomy done as per the study centre treatment protocol and the histology of the excised thyroid gland was noted. For both ultrasound and FNAC evaluation of thyroid masses in this study the variables of interest were the presence or absence of benign features or features suspicious of malignancy using the Bethesda system for reporting thyroid cytology [13].

**Data collection**: relevant socio-demographic data were documented with the aid of a proforma designed for the study. Data obtained from the study proforma, sonographic and cytologic patterns of the thyroid nodules were entered into a Microsoft office excel database. The sample size of 100 was estimated using the formula:

$${\text{N=[Z}}^{\text{2}}{\text{P(1-P)/d}}^{\text{2}}\text{]}$$

Where Z is standard normal variant of 1.96 at 95% confidence interval, P was the prevalence of 1.6 from a related study by Ogbera *et al*. [1], d the precision level was 0.025(2.5%). This gave the sample size, N as 96.77 which was rounded up to 107 to cover for possible attrition. Therefore data were gotten from 107 women.

**Statistical analysis**: was done using the Statistical Package for Social Sciences, version 20 (SPSS Chicago IL, USA). Continuous measurements was summarised in mean and standard deviation while categorical data were documented using frequency and percentages. Sensitivity, specificity, positive and negative predictive values were calculated to determine the diagnostic accuracy using 2-by-2 contingency tables.

**Ethical considerations**: written informed consent was obtained from all the study participants and privacy and confidentiality of patient´s data were ensured. Approval for the study was obtained from the Ethical and Research Committee of the Lagos State University Teaching Hospital with reference number LREC/10/06/607.

## Results

**Socio demographic characteristics of participants**: one hundred and seven (107) eligible participants with thyroid masses had both ultrasound and FNAC of their thyroids done and were analyzed. Ninety-one (85%) were females and 16 (15%) were males with female to male ratio of 5.7: 1>. The subjects were in the age range of 20-80 years. Majority were in the 41-50yrs age group with a mean age of 46.59 ±13.55 years (Table 1).

**Table 1:** socio-demographic characteristics of respondents

Variable	Frequency (n=107)	Percentage (%)
**Gender**		
Male	16	15.0
Female	91	85.0
**Age group (years)**		
21-30	14	13.1
31-40	23	21.5
41-50	33	30.8
51-60	23	21.5
>60	14	13.1
Mean± SD	46.59±13.55	
**Level of education**		
None	2	1.9
Primary	51	47.7
Secondary	37	34.6
Tertiary	17	22.8
**Occupation**		
Housewife	11	10.3
Business/Artisan	53	49.5
Civil servant	40	37.4
Retired	3	2.8

**Figure 1:**
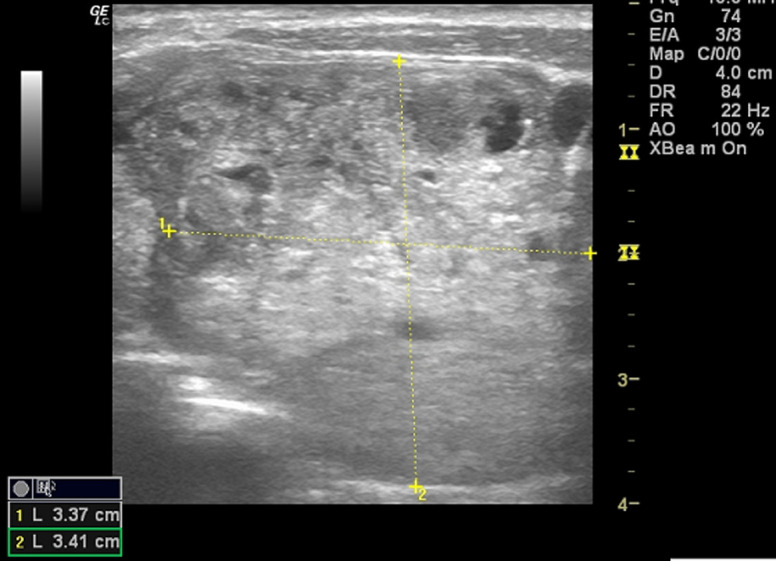
a predominantly solid thyroid nodule with irregular margins

**Figure 2:**
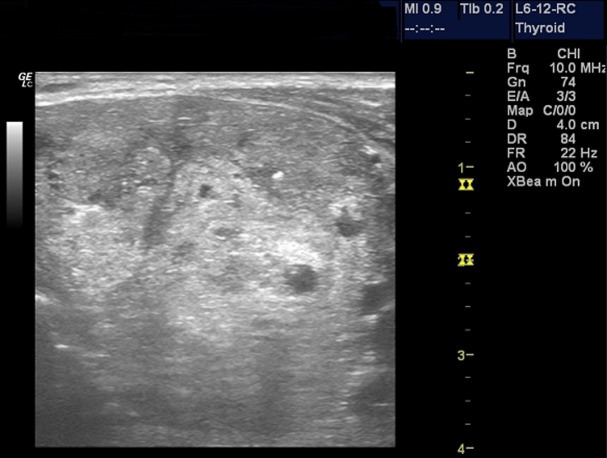
a heterogeneous nodule with punctate calcification

**Figure 3:**
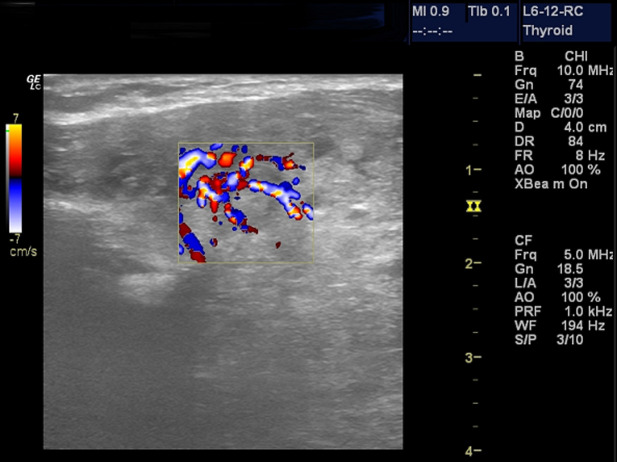
a thyroid nodule with central vascularity

**Sonographic characteristics of thyroid masses**: the location of the nodules was centre of the lobes in 54 (50.5%), 34 (17.8%) was on the right and 19 (31.8%) on the left. Eighty (74.8%) of the nodules were round, 25 (23.4%) were oval and 2 (1.9%) were irregular in shape. Ninety eight (91.6%) of the nodules had regular margins and 9(8.4%) had irregular margins (Figure 1). One hundred (93.5%) were of mixed echo-texture, 3 (2.8%) were hyperechoic, 2 (1.9%) were hypoechoic, and 2 (1.9%) were anaechoic (cystic). There was no calcification in the thyroid nodule in 101 (94.4%) patients and of those who presented with calcification, 5 (4.7%) were coarse calcification (Figure 2) and 1 (0.9%) had fine calcification. The echopattern of the nodule was heterogenous in 104 (97.2%) and 3 (3.8%) were homogenous (Table 2). On Doppler interrogation 75 (70.1%) of the nodules showed peripheral vascularisation, 22(20.6%) were avascular and 10 (9.3%) showed central vascularisation (Figure 3). Surrounding lymph node enlargement was present in 6 (5.6%) of the patients. The presence of thin peripheral halo around the nodules was demonstrated in 83 (77.6%). None of the thyroid nodules showed infiltration into surrounding structures (Table 2).

**Table 2:** sonographic features of the thyroid nodules

		Frequency (n=107)	Percentage (%)
**Shape**	Round	80	74.8
	Oval	25	23.4
	Irregular	2	1.9
**Margin**	Regular	98	91.6
	Irregular	9	8.4
**Echo-texture**	Hyper echoic	3	2.8
	Hypo-echoic	2	1.9
	mixed	100	93.5
	Anechoic	2	1.9
**Homogeneity**	Homogenous	3	3.8
	Heterogonous	104	97.2
**Calcification**	Fine	1	0.9
	Coarse	5	4.7
	Absent	101	94.4
**Vascularity**	Peripheral	75	70.1
	Internal	10	9.3
	Avascular	22	20.6
**Lymphadenopathy**	Present	6	5.6
	Absent	101	94.4
**Thin peripheral halo**	Present	83	77.6
	Absent	24	22.4
**Infiltration of adjacent muscles**	Present	0	0
	Absent	107	100.0

**Cytologic features of thyroid masses on FNAC**: all the 107 nodules analysed were found to be benign on FNAC (Table 3).

**Table 3:** features of thyroid nodules on FNAC

Features of thyroid nodules on FNAC	Frequency (n=107)	Percentage (%)
Cluster of follicular cell	102	95.3
Presence of thick colloid	98	91.6
Normal cytoplasmic ratio	79	73.8
Foamy microphages	65	60.7
Inflammatory cell	44	41.1
Nucleus contour round/ Oval	94	87.9
Presence of lymphocyte	46	43.0
Dense Nucleus	62	57.9

**Accuracy of thyroid sonography in predicting thyroid malignancy**: thyroid ultrasonography predicted benign thyroid lesions with an accuracy of 100%, specificity, sensitivity and positive and negative predictive value of 100% and negative predictive value of 10.0% when assessed with the FNAC and histology results combined (Table 4). The 2 patients identified with thyroid features as suspicious for malignancy on ultrasound were confirmed to be indeed malignant on histology but misclassified by FNAC as benign.

**Table 4:** accuracy of thyroid sonography in predicting thyroid malignancy

n=107	Histology + Cytology	
	Malignant	Benign
**Sonography**		
Malignant	TP=2	FP= 0
Benign	FN=0	TN=105

TP=True positive, TN=True negative, FP=False positive, FN=False negative, Sensitivity= TP / (TP + FN) = 100%, Specificity= TN / (FP + TN) = 100%, Prevalence of malignancy = 1.87%, Positive Predictive Value = TP / (TP + FP) = 100.00%, Negative Predictive Value = TN/(FN + TN) = 100.00%,Accuracy= (TP + TN)/(TP + TN + FP + FN) = 100%

## Discussion

The mean age of participants in our study is 46.59 ±13.55 years. Majority of the study participants with thyroid nodules were in the 41-50 years age group and female, with a female: male ration of 5.7: 1. This is similar to the study done by Ogbera *et al*. [1] in Lagos, Nigeria where the female to male ratio was 5: 1 and the mean age was 40± 12.4 years. A study done by Alam *et al*. [14] in Pakistan, of the 100 study participants 76 were females and 24 were males and the mean age was 41.8±12.3 years. These suggest that thyroid nodules are commoner in females.

Majority of the nodules 105(98.1%) exhibited strictly benign sonographic features of thyroid nodules which included well defined shape, regular margin, presence of thin peripheral halo, heterogeneity, absence of lymphadenopathy, peripheral vascularisation similar to previous studies [7]. Popli *et al*.in Delhi, India found that malignant nodules had irregular, lobulated margins, solid component and none of the nodules with cystic or predominantly cystic composition were found to be malignant[8]. Of the 107 nodules evaluated by ultrasonography, 2 showed features suspicious for malignancy which include presence of micro calcification, irregular outline and central vascularisation. The cytology report came out benign for all the 107 patients. The two patients with suspicious nodules on ultrasound eventually had surgery and histology of the mass confirmed malignancy. This is similar to study done by Vinayak and Sande [7] in Nairobi, Kenya where a total of 284 nodules were evaluated and the 234 labelled benign on USS were also confirmed on cytology as benign. Twenty of the fifty suspicious nodules on USS were malignant on cytology/histology. The most significant ultrasound characteristics used were margins, vascularity, echo-texture and presence of calcification [7]. Ancillary characteristics like shape of the nodule, presence of thin peripheral halo, lymphadenopathy, and infiltration into adjacent structure were not included in their study [7]. Recent reports indicate that studies of blood flow patterns are highly sensitive and specific in differentiating benign from malignant nodules [11,12]. The risk of malignancy increasing as intranodular blood flow becomes more dominant can be explained by the large cellular proliferations in this region [11,12]. Salehi *et al*. [12] found that on color Doppler sonography, central hypervascularity was the most common finding in malignant nodules while perinodular hypervascularity was the most common finding in benign nodules [12].

We found that ultrasound evaluation of the thyroid gland had sensitivity specificity and accuracy of 100% and positive and negative predictive value was 100% in predicting malignancy. Malik *et al*. [15] in a related study found a sensitivity, specificity, positive predictive value, negative predictive value and accuracy of 70.59%, 96.39%, 80%, 94.12% and 92% respectively when sonographic diagnosis was compared with FNAC in determining benign and malignant nodules [15]. Similarly in a study conducted by Popli *et al*. [8] the sensitivity, specificity, positive and negative predictive value for ultrasound diagnosis of benign and malignant thyroid nodules were 81.8%, 87.2%, 59.0% and 95.5% respectively [8]. Similar findings were obtained from study done by Ram *et al*. [16]. We found that ultrasound characterization of a thyroid nodule may accurately infer its benign nature since the 105 classified as benign was confirmed at FNAC. Furthermore, the two suspicious nodules on ultrasound suggested by FNAC to be benign eventually turned out to be malignant on histology.

## Conclusion

Ultrasound evaluation has a high diagnostic accuracy in predicting benign or malignantnature of thyroid nodules in African populations. Sonography can therefore be reliably used to assess for benign or malignant features of thyroid nodules and considered to replace the invasive assessment by FNAC in the face of current widespread challenges with pathologic reviews in many African health institutions.

**Limitations**: FNAC done in the study was not Ultrasound guided and may explain the reported discrepancy between FNAC and histology result.

**Recommendations**: sonographic assessment of thyroid masses without sequential FNAC even when malignant features are suggested can be reliably employed in the African population.

### What is known about this topic

- Sonography can be used to assess for benign or malignant features of thyroid nodules;
- Fine Needle Aspiration Cytology can be used to assess thyroid nodules.

### What this study adds

- Sonography can also be used to reliably assess for benign or malignant features of thyroid nodules and replace the invasive assessment by FNAC in black African populations;
- In highly suspicious malignant features, FNAC can be done under USS guidance to increase the diagnostic accuracy.
